# Case Report: Chronic subdural hematoma secondary to primary central nervous system lymphoma

**DOI:** 10.3389/fsurg.2025.1593112

**Published:** 2025-09-12

**Authors:** Xinli Mu, Zhihui Song, Qihong Wang, Hongsheng Yue, Xin He

**Affiliations:** ^1^ Department of Rehabilitation Medicine, Central Hospital Affiliated to Shandong First Medical University, Jinan, China; ^2^ Department of Neurology, Central Hospital Affiliated to Shandong First Medical University, Jinan, China

**Keywords:** chronic subdural hematoma (CSDH), primary central nervous system lymphoma (PCNSL), diffuse large B-cell lymphoma (DLBCL), craniotomy, case report

## Abstract

A 69-year-old man presented with a 15-day history of right-sided motor impairment and slurred speech. Twenty-one days earlier, he was misdiagnosed at a local hospital with a routine chronic subdural hematoma (CSDH) and underwent burr-hole drainage, but his symptoms progressively worsened postoperatively, leading to aphasia and prompting his admission to our hospital. Further MRI and contrast-enhanced imaging revealed hematoma organization, brain herniation, and an intracranial mass lesion. The patient underwent craniotomy for tumor resection and evacuation of the organized hematoma. The patient received structured rehabilitation and limb positioning therapy during hospitalization to support motor recovery and prevent complications, and was discharged on postoperative day 13 with improved limb function but persistent aphasia. Histopathological analysis confirmed non-germinal center diffuse large B-cell lymphoma (DLBCL) within the hematoma, supported by immunohistochemical and FISH findings, including CD20(+), PAX-5(+), MUM-1(+), and Ki-67(+, 60%). The patient underwent four cycles of rituximab and high-dose methotrexate, resulting in lesion resolution on follow-up MRI, with motor aphasia persisting. This case represents a rare instance of primary central nervous system lymphoma (PCNSL) initially presenting as CSDH, with progression to hematoma organization and brain herniation. This case provides new insights and experience in recognizing and managing rare clinical presentations of primary central nervous system lymphoma.

## Introduction

Primary central nervous system lymphoma (PCNSL) is a rare extranodal non-Hodgkin lymphoma that affects the brain, spinal cord, leptomeninges, or vitreoretinal region, typically in the absence of systemic disease. Its diagnosis is often delayed due to highly variable clinical manifestations, which depend on the location of the lesion (1). In atypical cases, early recognition may be further complicated when symptoms resemble more common conditions, such as chronic subdural hematoma (CSDH). CSDH is a frequently encountered neurosurgical condition in elderly patients, most often linked to minor head trauma or coagulopathy (2). We describe a rare case of PCNSL that initially mimicked CSDH and progressed to hematoma organization, ventricular deformation, and brain herniation—ultimately requiring emergency craniotomy for diagnosis and treatment.

## Case report

In August 2023, a 69-year-old male patient from China presented to the hospital with a 15-day history of impaired movement in the right limbs accompanied by slurred speech. Half a month prior to admission, the patient developed right-sided motor dysfunction and speech impairment, without accompanying symptoms such as headache, dizziness, nausea, vomiting, seizures, or bowel and bladder incontinence. Since the onset of symptoms, the patient remained conscious but exhibited poor mental status. His appetite and sleep were normal, and bowel and bladder functions were unaffected. He denied any history of major internal diseases such as hypertension or coronary artery disease.

**Figure 1:**
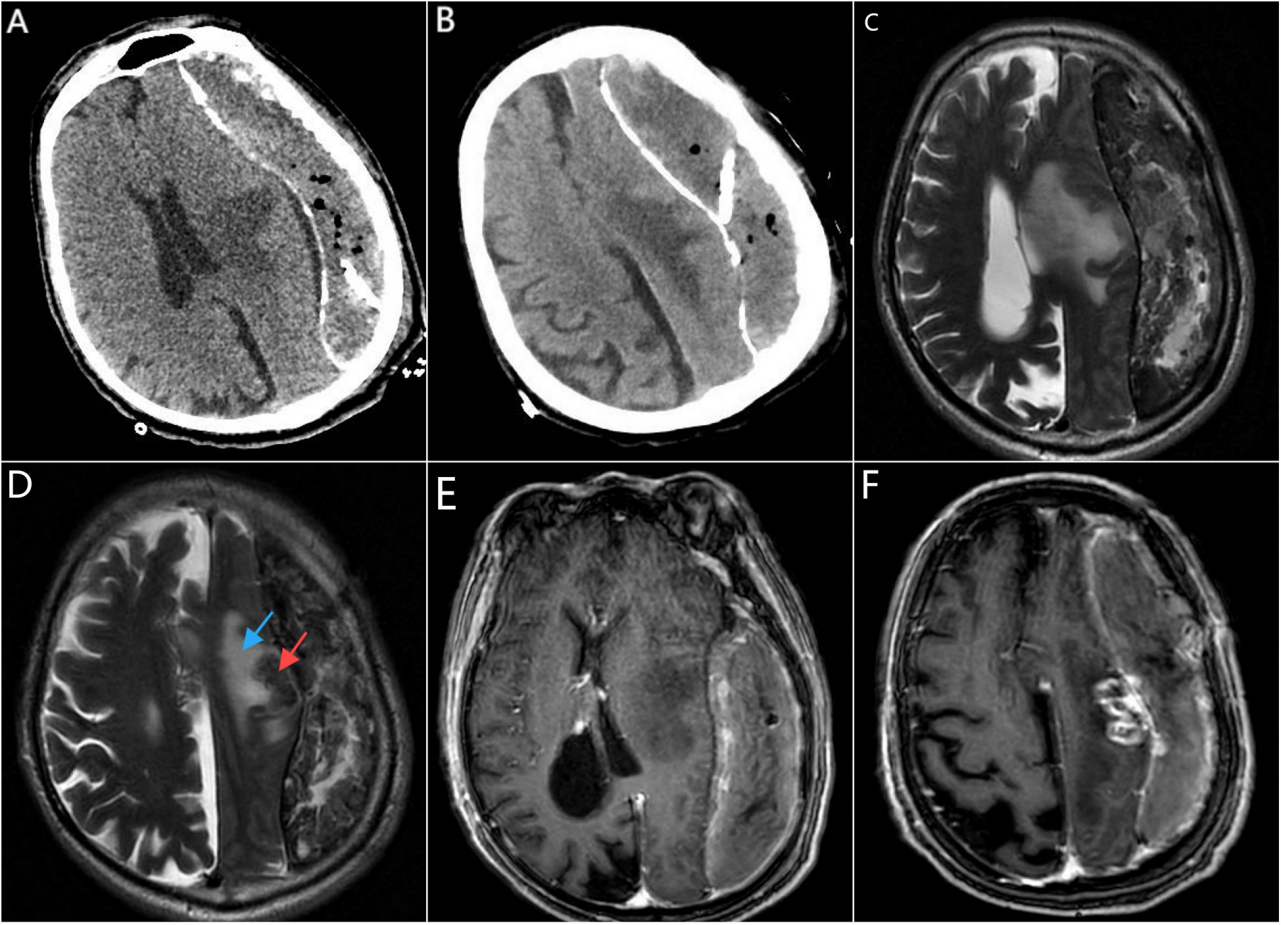
CT, MRI, and contrast-enhanced MRI imaging prior to intracranial tumor resection. **(A,B)** were obtained on the first day after burr-hole drainage; **(C–F)** on day 21 after burr-hole drainage. **(A)** Axial cranial CT shows a drainage tube, compression of the left cerebral hemisphere parenchyma, and a hypodense lesion in the left basal ganglia (cause unclear). **(B)** Axial cranial CT reveals a hypodense lesion in the left centrum semiovale. **(C)** Cranial MRI (Propeller T2, oblique axial) on postoperative day 21 indicates a chronic subdural hematoma on the left side, a mass lesion in the left centrum semiovale, and significant midline shift. **(D)** Cranial MRI (Propeller T2, oblique axial) demonstrates a round isointense lesion (red arrow) in the left centrum semiovale surrounded by a hyperintense T2 edema zone (blue arrow). **(E)** Contrast-enhanced cranial MRI (sT1W 3D IR TFE, axial view) shows no enhancement of the hypodense lesion in the left basal ganglia. **(F)** Contrast-enhanced cranial MRI (sT1W 3D IR TFE, axial view) reveals a ring-enhancing lesion in the left centrum semiovale, suggestive of a metastatic tumor.

Twenty-one days prior to admission, the patient experienced an unexplained fall followed by slurred speech. A cranial Computed Tomography (CT) performed at a local hospital revealed a chronic subdural hematoma in the left frontal, temporal, and parietal regions. The patient underwent burr-hole drainage for the subdural hematoma. A follow-up CT on the first postoperative day showed a strip-like mixed hypodense shadow with calcified edges under the inner plate of the left frontal, temporal, and parietal bones, containing gas shadows and the drainage tube. The left cerebral hemisphere parenchyma appeared compressed. Patchy hypodense lesions were noted in the left basal ganglia and centrum semiovale, with clear boundaries. Mild ventricular enlargement was observed bilaterally, with widened sulci and fissures. The midline structures were shifted to the left (Figures 1A,B). Considering the hypodense lesions in the left basal ganglia and centrum semiovale, the local hospital recommended further imaging, including Magnetic resonance imaging (MRI), which the patient's family declined. The symptoms of aphasia and limb motor function gradually worsened 7 days after burr-hole drainage.

The patient denied any history of smoking, alcohol consumption, or illicit drug use, and there was no reported family history of hereditary diseases or malignancies.On physical examination, the patient was unable to communicate and exhibited slurred speech, aphasia, and poor mental status. No other significant abnormalities were observed. The patient followed instructions and cooperated during the examination. Both pupils were equal in size and round, with a diameter of approximately 2.5 mm, and reactive to light. No noticeable nasal swelling or active bleeding was identified. The nasolabial folds were symmetrical, the tongue was midline, and no nuchal rigidity was noted. Muscle strength and tone in all four limbs were within normal limits, and Babinski signs were bilaterally negative. Laboratory investigations showed no abnormal findings. Routine blood tests, liver function tests, and tumor markers, including CEA, CA19-9, CA-125, and AFP, were all within normal ranges.

**Figure 2:**
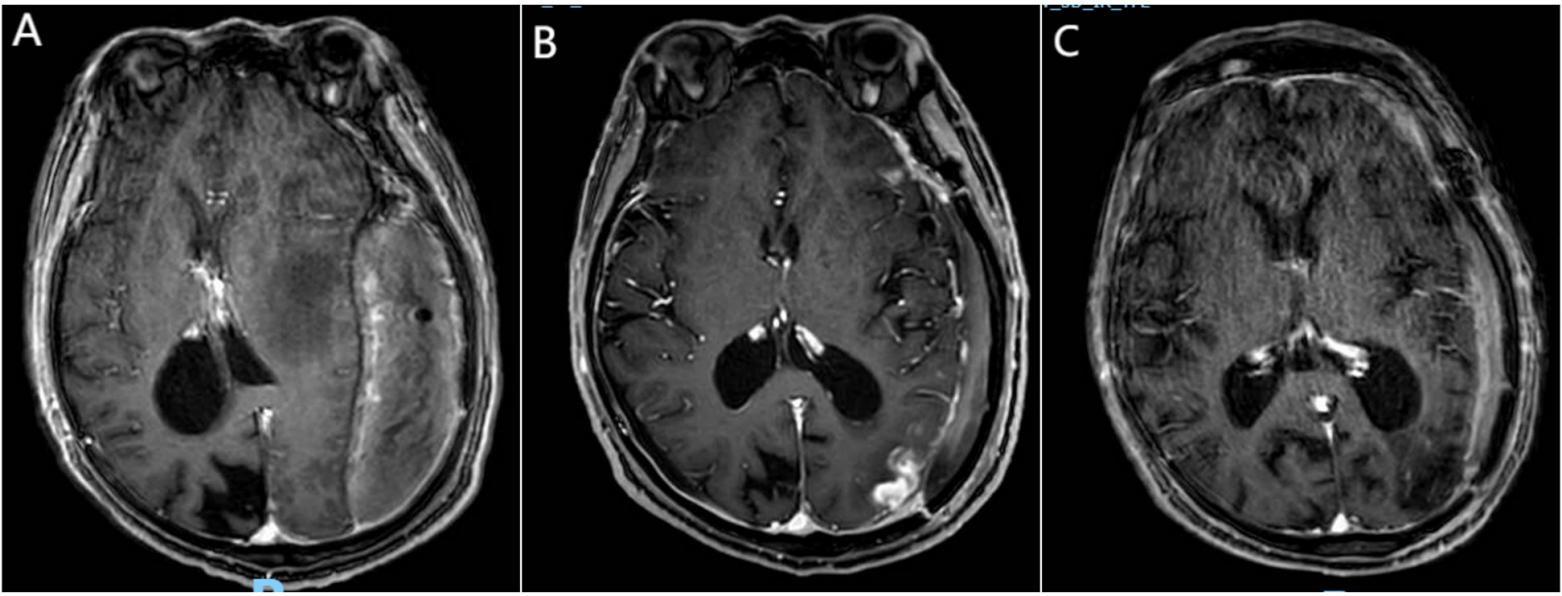
Newly identified lymphoma lesion. **(A)** Preoperative contrast-enhanced MRI (sT1W 3D IR TFE, axial slice through lateral ventricle body) shows no enhancement in the left occipital subcortical region, indicating no evidence of new lymphoma lesion preoperatively. **(B)** Contrast-enhanced MRI (sT1W 3D IR TFE, axial view) 13 days postoperatively reveals a new, irregularly enhanced lesion in the left frontal subcortical region, considered to be lymphoma. **(C)** Contrast-enhanced MRI (sT1W 3D IR TFE, axial view) after four cycles of chemotherapy shows complete resolution of the newly identified irregularly enhanced lesion in the left frontal subcortical region.

MRI of the brain, including plain and diffusion-weighted imaging (DWI), revealed a strip-like mixed T1 and T2 signal shadow measuring approximately 15 × 3.7 cm beneath the inner table of the left frontotemporal skull. A small amount of gas signal was present within the lesion. The DWI showed a mixed signal pattern, and the apparent diffusion coefficient (ADC) displayed areas of reduced signal intensity. The brain parenchyma of the left cerebral hemisphere was compressed, with a significant midline shift to the right. A round isointense lesion was observed in the left centrum semiovale, surrounded by patchy areas of long T1, long T2, and high FLAIR signals indicative of edema. Radiological impressions suggested a chronic subdural hematoma on the left side with significant midline shift, raising concerns for brain herniation, and a mass lesion in the left centrum semiovale (Figures 1C,D). Subsequent contrast-enhanced brain MRI showed an irregular mixed signal lesion in the subcortical region of the left frontal lobe, measuring approximately 26 × 13 mm. The lesion exhibited ring-like enhancement with perilesional edema. Additionally, uneven enhancement of the left meninges was noted. The strip-like mixed signal shadow beneath the inner table of the left frontotemporal skull showed heterogeneous enhancement and contained a small amount of gas signal. The brain parenchyma of the left cerebral hemisphere and the left lateral ventricle were compressed, with a significant midline shift to the right. Radiological impressions suggested a subcortical lesion in the left frontal lobe that could be a metastatic tumor. Chronic subdural hematoma on the left side with brain herniation was also considered (Figures 1E,F–2A). Based on the patient's medical history, physical examination, laboratory findings, and imaging studies, the diagnosis of a left frontoparietal lesion accompanied by progressive chronic subdural hematoma with evidence of hematoma organization and brain herniation was established.

**Figure 3:**
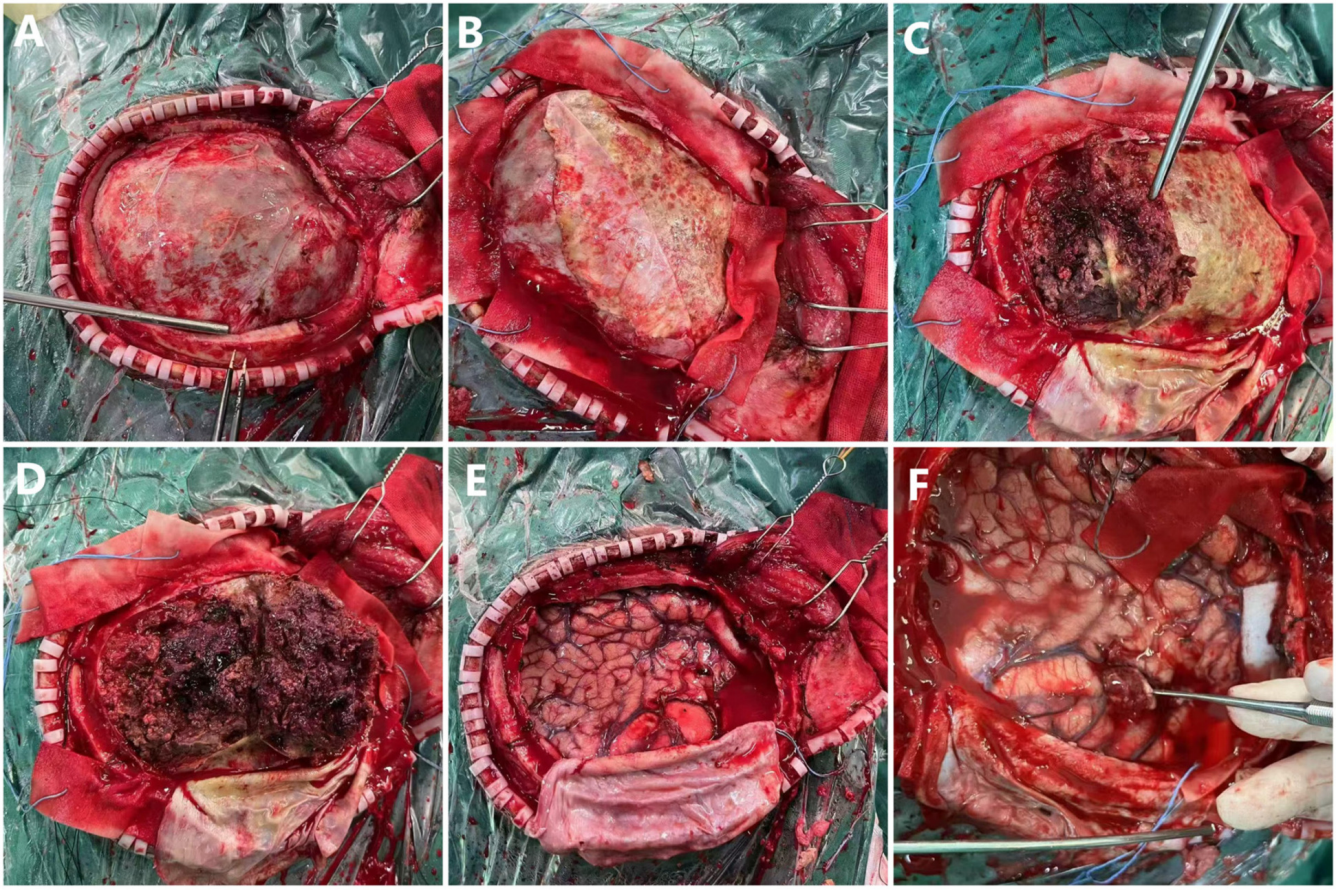
Intraoperative photographs of craniotomy. **(A)** Exposed dura mater. **(B)** Dura mater partially retracted to the left, revealing organized hematoma. **(C,D)** Hematoma being removed. **(E)** Exposed brain tissue after complete hematoma removal. **(F)** Identified mass lesion.

The patient underwent left intracranial tumor resection, evacuation of the subdural hematoma, and removal of the hematoma capsule under general anesthesia. A modified pterional approach was performed, with five burr holes drilled to create a bone flap approximately 12 × 10 cm in size at the left frontotemporal region. Upon opening the dura, it was found to be tense and hardened. After incision and separation, the hematoma appeared fully organized, gray-brown in color, and tofu-like in texture. The brain tissue was significantly compressed, with no pulsations observed. Approximately 250 ml of organized hematoma and hematoma capsule were evacuated, after which brain pulsations were restored. A tumor approximately 2.5 × 2.6 cm in size was then resected. The tumor was gray-brown, firm, and poorly demarcated (Figure 3). No active bleeding was noted. There was no significant elevation of intracranial pressure. Decompressive closure is generally not required in standard CSDH procedures. An artificial dura was used to repair the dura in a decompressive manner. Surgical instruments and gauze counts were verified, and a subdural drainage tube was placed. Standard closure of the cranial cavity was performed. The resected tumor and hematoma capsule were sent for pathological analysis.

**Figure 4:**
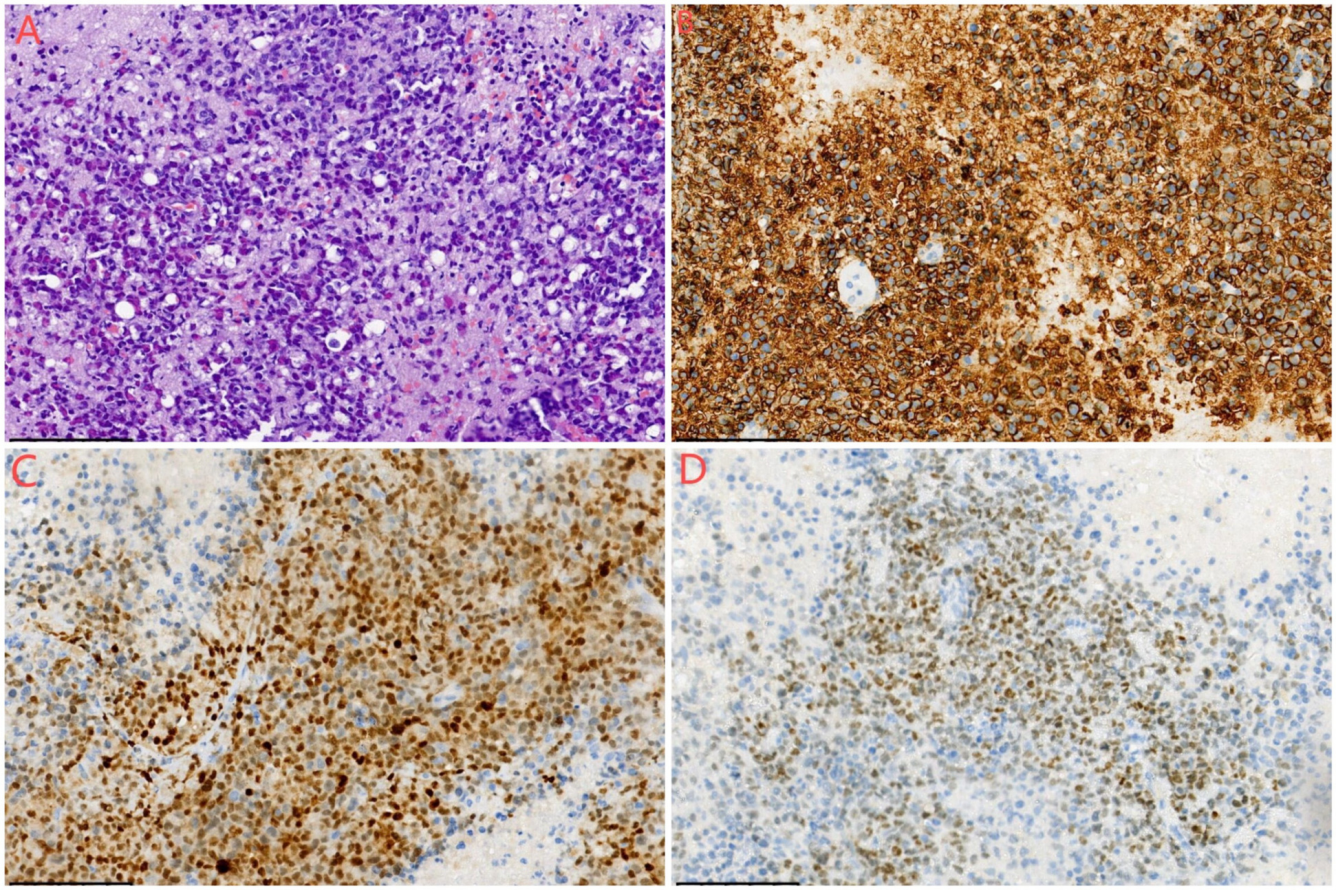
Histological examination of the mass lesion. **(A)** Hematoxylin and eosin (HE) staining, ×200 magnification. The morphology indicates high cellularity, with large tumor cells featuring round or ovoid nuclei, dense chromatin, distinct nucleoli, and diffuse, patternless growth. **(B)** Immunohistochemistry, ×200 magnification, showing CD20(+) staining. **(C)** Immunohistochemistry, ×200 magnification, showing MUM-1(+) staining. **(D)** Immunohistochemistry, ×200 magnification, showing PAX5(+) staining.

Gross pathological examination revealed multiple fragments of cyst wall-like tissue from the left subdural hematoma and its capsule, measuring 10 × 5 × 2 cm, with a wall thickness of 0.1–0.3 cm and a gray-red, firm texture. The frontal lobe lesion consisted of multiple fragments of gray-red, soft tissue measuring 3 × 2.5 × 1 cm. The morphological indicated that the tumor exhibited high cellularity, with large tumor cells possessing round or ovoid nuclei, dense chromatin, and distinct nucleoli, displaying diffuse and patternless growth. Examination of the subdural hematoma and its capsule revealed extensive coagulated blood clots and necrotic tissue beneath the fibrous capsule, along with a small amount of plasma cell infiltration. Immunohistochemical and FISH testing results staining showed CD20(+), PAX-5(+), MUM-1(+), C-Myc (+, approximately 40%), CD31(−), PTEN(−), S-100(−), GFAP(−), EMA(-), CK(−), CD138(−), CD38(−), CD3(−), CD10(−), CD79a(−), CD5(−), Bcl-2(−), Bcl-6(−), P53(+, approximately 10%), and Ki-67(+, approximately 60%) in the frontal lobe lesion (Figure 4). The morphological, immunohistochemical, and FISH testing results the frontal lobe lesion was consistent with non-germinal center type diffuse large B-cell lymphoma (DLBCL).

The patient was discharged 13 days postoperatively with well-healed incisions. While some improvement in limb motor function was observed, there was no significant recovery in aphasia. During hospitalization, the patient underwent structured rehabilitation education and functional training under the guidance of ward medical staff, with a particular focus on proper limb positioning to promote recovery and prevent complications. Good limb positioning techniques were implemented, including multiple posture-based interventions. In the side-lying position with the hemiplegic side down, the shoulder was protracted by 45° to reduce compression, and the hip and knee joints were slightly flexed. Training for the non-hemiplegic limb included arm swings, wrist rotations, and finger exercises—five repetitions per set, ten sets per day. Subsequently, the patient was repositioned with the hemiplegic side up, supported with pillows for comfort, and engaged in the same training regimen for the affected limb to preserve mobility. In the supine position, thin pillows supported the hemiplegic shoulder and hip, with the arm placed in external rotation and abduction, and fingers extended to prevent contractures. Seated training involved upright posture in bed or in a wheelchair, with the hemiplegic arm supported and extended on a tray or pillow, and lower limbs positioned to maintain joint alignment and prevent foot drop. These comprehensive positioning and rehabilitation measures aimed to enhance functional recovery and minimize long-term disability.

On the day of discharge, an enhanced MRI revealed a new high-density lesion on the left side, considered to be lymphoma (Figure 2B). The patient subsequently continued chemotherapy and rehabilitation therapy. Over a five-month follow-up period, the patient received two cycles of chemotherapy with rituximab combined with high-dose methotrexate. Follow-up cranial MRI showed a slight reduction in the size of the new lesion, while no recurrence was observed in the lesion that had been surgically removed. After an additional two cycles of rituximab combined with high-dose methotrexate, the new lesion disappeared (Figure 2C). Chemotherapy was continued. At the most recent follow-up, the patient exhibited persistent motor aphasia, but the right limb motor function was almost normal, and no other neurological deficits were noted.

## Discussion

PCNSL is a rare subtype of non-Hodgkin lymphoma that primarily involves the brain, leptomeninges, spinal cord, and/or eyes, without evidence of systemic disease (3). PCNSL accounts for approximately 4% of all intracranial tumors and 4%–6% of all extranodal lymphomas, with a higher incidence observed in immunocompromised individuals. Over 90% of PCNSL cases are DLBCL, predominantly of the non-germinal center subtype (4, 5). PCNSL-DLBCL typically expresses B-cell antigens such as CD79a, CD19, CD20, and surface immunoglobulin light chains. Genetic mutations, including MYD88 mutations, CDKN2A biallelic deletions, and CD79B mutations, are frequently observed. Rearrangements of the BCL-6 gene are associated with a worse prognosis. Additionally, PCNSL can manifest as other subtypes, such as Burkitt lymphoma, indolent B-cell lymphoma, or *T*-cell lymphoma (6).

PCNSL presents with a diverse range of neurological symptoms or signs depending on the affected site. These may include focal neurological deficits (e.g., paralysis, ataxia, gait disturbances, or speech disorders), progressive cognitive impairment, behavioral changes, symptoms of increased intracranial pressure (e.g., headache, nausea, vomiting, papilledema), and seizures. In rare cases, patients may develop parkinsonian syndromes (7). When PCNSL involves the subependymal region, it may present with periventricular nodules, extending to structures such as the choroid plexus, corpus callosum, pituitary gland, and pineal gland, leading to endocrine disorders, cranial nerve deficits, or ataxia (7). Obstruction of cerebrospinal fluid pathways can result in hydrocephalus and symptoms of intracranial hypertension. PCNSL may also involve small cerebral vessels, causing symptoms resembling lacunar infarctions or subcortical dementia and increasing the risk of venous thrombosis and hemorrhage (8). In addition to the brain, lymphoma cells may infiltrate the spinal cord, cerebrospinal fluid, or eyes, resulting in corresponding clinical manifestations (1).

This case is unique in that CSDH was the initial clinical presentation. Chronic subdural hematoma is a common neurosurgical condition in the elderly, with an estimated annual incidence of 1.7–20.6 cases per 100,000 (9). Risk factors include cerebral atrophy, altered parenchymal compliance, coagulopathy, immune system dysregulation, and head trauma (9, 10). However, cases of chronic subdural hematoma caused by primary central nervous system lymphoma are exceedingly rare. Katsuharu Kameda (11) reported a case of a 96-year-old man with an organized chronic subdural hematoma (OCSH) in the left frontal convexity and a *de novo* mass beneath it on MRI. Surgical removal of the hematoma and tumor revealed Epstein–Barr virus-positive DLBCL in both the brain parenchyma and the OCSH, suggesting the lymphoma originated in the hematoma and infiltrated the brain. Yu et al. (10) described an 82-year-old patient with a two-day history of headache and consciousness disorder after head trauma. Initially misdiagnosed as a subacute epidural hematoma, craniotomy revealed a subdural lesion, which was later confirmed as subdural anaplastic large-cell lymphoma (SALCL) by histopathology. Unfortunately, the patient died one month after discharge.

Given the patient's overall good health, absence of coagulopathy, immune dysfunction, or trauma history, and the pathological confirmation of PCNSL, it is likely that the chronic subdural hematoma resulted from lymphoma-related mechanisms such as tumor infiltration of the dura or leptomeninges, vascular fragility, or spontaneous microhemorrhage induced by malignant involvement. In retrospect, performing only burr hole drainage for the subdural hematoma during the patient's initial presentation was inappropriate. The rarity of lymphoma as the underlying cause and the incomplete diagnostic workup led to an unclear diagnosis at the time, making the decision for burr hole drainage at the local hospital hasty. Additionally, the hematoma had already become organized, and simple burr hole drainage was insufficient; urgent craniotomy for hematoma evacuation should have been performed instead. However, the responsibility cannot solely be attributed to the physicians. At the initial presentation, the patient and their family, due to financial constraints, refused further diagnostic or therapeutic interventions. Ultimately, the delay in definitive treatment for two weeks resulted in disease progression and complications, including aphasia. This case underscores the challenges in diagnosing primary central nervous system lymphoma while offering a comprehensive account of its treatment course.

From clinical experience, combination chemotherapy has demonstrated excellent efficacy in the treatment of lymphoma. However, the unique anatomical location of PCNSL and the presence of the blood-brain barrier limit the effective intratumoral concentration of certain chemotherapeutic agents. High-dose methotrexate-based regimens are currently the standard induction therapy for PCNSL (12). Surgical intervention has minimal impact on overall survival (OS) and progression-free survival (PFS) and may lead to severe postoperative complications such as brain herniation or neurological damage (1, 3). Consequently, surgery is typically limited to biopsy or urgent decompression in PCNSL patients. Although surgical resection provides a pathological diagnosis, it is not a primary treatment modality. Unlike other malignant lymphomas, PCNSL often involves deep structures and extensive lesions, making complete resection unfeasible (13). In recent years, stereotactic biopsy has emerged as a preferred alternative to craniotomy due to its minimal invasiveness, higher success rate, and repeatability, enabling the acquisition of pathological diagnoses while minimizing patient discomfort. For patients with radiological findings suggestive of PCNSL on CT or MRI, stereotactic biopsy is recommended to confirm the diagnosis (1). In this case, craniotomy was performed due to the rare complication of chronic subdural hematoma, its organized state, and the development of brain herniation, necessitating emergency intervention.

## Conclusion

PCNSL presenting as CSDH with brain herniation is extremely rare and easily misdiagnosed, particularly in the absence of trauma or coagulopathy. In this case, the initial diagnosis of CSDH led to delayed identification of the underlying malignancy, ultimately requiring emergency surgical intervention. This case highlights the importance of considering alternative or atypical causes of CSDH when conventional risk factors are absent. Early use of contrast-enhanced MRI and timely pathological evaluation are essential for accurate diagnosis and appropriate management in such atypical presentations.

## Data Availability

The original contributions presented in the study are included in the article/Supplementary Material, further inquiries can be directed to the corresponding author.
